# Investigating the Potential Role of Ecological Validity on Change-Detection Memory Tasks and Distractor Processing in Younger and Older Adults

**DOI:** 10.3389/fpsyg.2019.01046

**Published:** 2019-05-24

**Authors:** Ulrike Rumpf, Inga Menze, Notger G. Müller, Marlen Schmicker

**Affiliations:** ^1^Neuroprotection Laboratory, German Center for Neurodegenerative Diseases, Magdeburg, Germany; ^2^Otto von Guericke University of Magdeburg, Magdeburg, Germany; ^3^Center for Behavioral Brain Sciences, Magdeburg, Germany

**Keywords:** cognitive control, selective attention, memory, distractor inhibition, aging, everyday behavior

## Abstract

Cognitive performance is often found to be lower in older adults, especially when the task requires memory, executive functions, or selective attention. But this alleged deterioration may have been overestimated in the past due to ecologically invalid testing. To verify this possible misjudgment here we compared age-related memory performance in a typical, abstract computer task to a paper-pencil test with a real-world map and to an even more realistic task that took place in a real room with everyday objects. Retention and response intervals differed between the tasks as they had to be adjusted to the different settings. Twenty-seven younger (19–29 years old) and twenty-three older participants (61–77 years old) took part in the study. As expected younger participants outperformed the older ones in the computer task. However, although older adults’ performance was better in both more realistic tasks, the delta to the young remained the same as in the computer task. Hence, these results do not support the general notion that older adults would profit from more realistic test scenarios. On the other hand, performance in a clinical screening task correlated only with the performance in the real world task suggesting that this task reflected the general cognitive status of participants better than the more abstract tasks. Finally, it was observed that the presence of task-irrelevant distractor items actually helped older adults to improve their performance in the paper pencil task arguing against the assumption of a general age-related impairment of inhibition. In sum, the present results show that age-related changes in memory are neither simply explained by reduced abilities to deal with abstract computer tasks nor by disturbed inhibition processes.

## Introduction

### Why Ecological Validity Is Especially Important for Aging

For many years, researchers have been discussing whether to focus on internal or external validity in experimental designs. The obtained findings of internally valid research enable an explicit conclusion respecting the causal influence of the manipulated variable to the outcome (Eid et al., 2010). Internally valid results therefore lack to reach generalizations to other places, people, times, and situations. In 1976 Ulric Neisser claimed that memory research of the last 100 years seemed to be worthless because it failed to answer questions for everyday life (Cohen, 1989).

So far, much effort has been put into achieving ecological validity in experimental task settings by developing more realistic tasks in and outside the laboratory. Tasks such as the virtual week studies (Rendell and Henry, 2009) or the Breakfast task (Craik and Bialystok, 2006b) are only a few. Especially, older adults could benefit from this development.

Aging in humans is accompanied by a decline in cognitive processes such as memory, executive functions and the related attentional processes, which are necessary for processing and maintaining a huge variety of information (Naveh-Benjamin, 2000; Craik and Bialystok, 2006a). However, aging differences in lab-based cognitive performance are not only predictable by cognitive decline. Older age is additionally characterized by reduced processing as well as decreased response speed in computerized experiments (Cherry and Park, 1993; Mazurek et al., 2015). As older adults need to use environmental and cognitive support to compensate for cognitive decline (Bäckman, 1985), test settings far from reality may not be suitable for memory strategies like chunking or organization of storage material. Besides using strategies in cognitive testing, the performance in older age is positively affected by three-dimensionality, contextual information, and multisensory processing. Crawford and Channon (2002) investigated problem-solving in younger and older adults and observed that older participants benefitted from their experience and pragmatics by producing less but qualitatively superior solutions. Cherry and Park (1993) compared younger and older adults’ performances in a 2D and 3D test setting and found that both age groups increased their performance in a 3D environment while age-related differences were reduced. Age differences in memory performance measured in a laboratory setting may be also a result of different features of the task. Younger adults may not give their best in experiments outside the lab (The Age Prospective Memory Paradox; Aberle et al., 2010). Therefore, ecologically valid testing is of particular importance to investigate age-related decline.

Some studies found that cognitive abilities in age seem to be unimpaired within realistic memory settings while the same task assessed as paper-pencil or computer task results in cognitive disadvantages for higher age (Mazurek et al., 2015). Reality-based tests could be more indicative of human behavior as they are closer related to experiences of older adults in everyday life (Craik and Bialystok, 2006b), but these tests are quite rare. There is a need for more ecological experiments reflecting real performances in older adults as computerized laboratory tests may fail to measure everyday-related cognitive constructs.

### Examining Age-Related Differences in Attentional Control

Executive functions, processing speed and episodic memory are of particular interest in the context of aging because they decline with age (Craik, 1994; Hedden and Gabrieli, 2004; Craik and Bialystok, 2006b). Especially working memory (WM), which is considered a basic mechanism that relates to a wide range of other cognitive functions (Johnson et al., 2013), is observed to decline with age (Salthouse, 1994; Chen et al., 2003). On an individual level, several studies indicated that humans with high WM capacity are better in controlling their attention. They are more capable of concentrating on relevant information and of filtering out irrelevant information (Vogel and Machizawa, 2004; Vogel et al., 2005). Moreover, the hypothesis of reduced attention resources (Craik and Byrd, 1982) assumes that older people have fewer resources for encoding and storing information available. Another attention-based theory claims that aging goes along with deficient inhibitory processes, especially for the ability to ignore irrelevant distractors (Lustig et al., 2007).

In order to address these age-related changes, a study investigated distractor inhibition in WM performance in younger and older adults (Jost et al., 2010). They reported decreased filtering in older adults compared to younger adults, but only early in the retention interval. Therefore, we focused on so-called change-detection tasks that usually investigate distractor inhibition^1^ in WM (Vogel and Machizawa, 2004; Vogel et al., 2005; Jost et al., 2010). Participants have to remember a fixed number of stimuli (e.g., bars or circles) and then detect a change in one attribute (e.g., color or orientation) after a delay. To induce interference effects, participants were presented additional, irrelevant stimuli. These distractors are different from targets and have to be ignored. Results show that additional distractors lead to lower memory performance (Vogel et al., 2005; Jost et al., 2010).

These computer-based change-detection tasks are highly internally valid and discover cognitive aging mechanisms by systematic manipulation, but may underestimate an individual’s cognitive ability in everyday life situations (Noyes and Garland, 2008). Some people are less experienced in handling the computer (e.g., clicking a mouse, using a keyboard, or response buttons), might have reduced processing speed as well as more stress with the encoding of abstract, non-realistic stimulus material used in computer tasks (Wästlund et al., 2005; Schmicker et al., 2016). These negative experiences in computerized paradigms frustrate older adults. Their insecurity, anxiety or inexperience might potentiate this effect (Colquitt et al., 2000; Czaja et al., 2006).

Therefore, these computerized change-detection tasks do not seem to be suitable for valid cognitive investigations. Laboratory-based realistic tasks can possibly provide the requirements for processing memory content more appropriately. On the one hand, adapting working memory experiments to real environments turns out to be very difficult because practical tasks come along with longer times for conducting the measurements. On the other hand, more time for processing information is closer to actual everyday behavior and could enable older adults to apply their cognitive reserve (Stern, 2002) or develop compensational strategies (Garrett et al., 2010), which result in successful memory and inhibition performance.

### Aims of the Study

In this study, we wanted to investigate the feasibility of measuring distractor processing in more realistic environments and compare it between younger and older adults. We devised three tasks based on the change-detection task that has shown the distractor inhibition effect in working memory performance.

First, we used a task measuring distractor inhibition in working memory from our earlier studies. This computerized task was a further development of the common Vogel-task that measures visual-spatial memory performance (Vogel and Machizawa, 2004; Vogel et al., 2005; Schmicker et al., 2016, 2017). Participants have to remember the position and orientation of rectangles of one color while rectangles of another color need to be inhibited (distractor inhibition, *DIIN*). The arrays of rectangles are presented in succession, either with or without distractors.

Second, we invented a task in a real room (*realDIIN*). Based on the computerized *DIIN* task (Schmicker et al., 2016), the task was to encode either red or green marked office objects while in some trials also distractors were present. Participants were able to move around freely and access contextual information of the test environment.

Third, we created an additional paper-pencil task, the *CityMap*, in order to verify the influences of computerized settings and additional context information. In this paper-pencil task, a map of the city Basel with red and green marked buildings was presented, offering many contextual reference points but no third dimension or the possibility of walking around.

We were also interested in motivational aspects of closeness to reality and the relationship to cognitive measures using a screening for mild cognitive impairment (The Montreal Cognitive Assessment, *MoCA*).

In this study, an aim was to identify age-related differences between internally and ecologically valid tasks. However, there were two difficulties: First, ecologically valid, realistic tasks are hardly comparable to original, internally valid computerized experiments. The arrangement of real environments requires different stimulus material (shapes in computerized tasks are not as realistic as real objects) and longer encoding/retention intervals (the examiner needs time to arrange the test setting and the participant has to process more complex stimuli). However, these changes are necessary to evoke the task-related knowledge which might be the cause for age-related compensation (Phillips et al., 2006). Second, in order to examine interaction effects between age groups and task types, we calculated the required sample size. For an assumed medium effect size, the computed *N* was 418. It was not realizable to test such a large sample size with three different tasks (considering that one measurement took 3 h). Therefore, we would like to handle this work as a feasibility study. In this regard, the results of age group × task type effects should be interpreted with caution.

We expected that all three tasks would be able to depict the distractor effect (worse performance when distractors were present), that more realistic test settings induce higher performance, higher motivation and a more pronounced relationship to cognitive screening measures. We also expected that older adults profit from attributes of more realistic test designs and are able to compensate for performance deficits by using their cognitive reserve.

## Materials and Methods

### Participants

In sum, 60 people were recruited, who were healthy, right-handed, showed no color blindness and had a correct or corrected to normal vision. Each participant received 35€ for participating.

Fifty six participants met the inclusion criteria and took part in the examination (four older participants failed to reach the required *MoCA* score of at least 26 points). Another six participants (three younger and three older ones) had to be excluded after outlier analysis because they performed extremely good or bad at one of the change-detection tasks (*M* ± 2*SD*). Thus they were considered as outliers to the whole sample and were excluded from all analyses. The results of 27 young participants aged between 19 and 29 years (*M* = 23.56 years, *SD* = 2.71, 14 women) and 23 older participants aged between 61 and 77 years (*M* = 69.78 years, *SD* = 4.07, 12 women) were finally analyzed. Concerning the years of education, younger (*M* = 15.91 years, *SD* = 2.56) and older adults (*M* = 16.11 years, *SD* = 2.88), did not differ significantly.

### Design and Procedure

The whole measurement was conducted in a single session that took 3 h. Each subject got informed about the study, the procedure, conditions of participation and the processing of data in the beginning. After signing the declaration of consent, demographic data were assessed (age, gender, handedness, years of education, diseases, medication, computer experience, average time spent on computers in a week). Second, the *MoCA* was used as a screening inventory (Nasreddine et al., 2005) to exclude cognitively impaired participants. Younger participants had to complete the *MoCA* as well in order to guarantee equality in test conditions and motivational aspects for younger and older. Afterward, the participants performed the *PC-DIIN*, *realDIIN*, and *CityMap*; the test order was counterbalanced across individuals. Immediately after each task, the participants filled out a task specific motivation questionnaire. After they had finished all tasks, there was a final motivational examination to compare the tasks retrospectively.

The study protocol was approved by the ethics committee of the University of Magdeburg (Germany) and all participants gave written informed consent in accordance with the Declaration of Helsinki.

### Material

This study used three types of tasks to investigate behavioral differences related to the degree of ecological validity.

#### Computer-Based Change-Detection Task (*PC-DIIN*)

The computer based task measuring working memory and distractor inhibition was developed by Schmicker et al. (2016) and adapted for this examination. The time intervals were elongated to keep older adults’ correct answers above chance level. Furthermore, the number of trials was changed and a mask followed by forced-choice retrieval was implemented.

**FIGURE 1:**
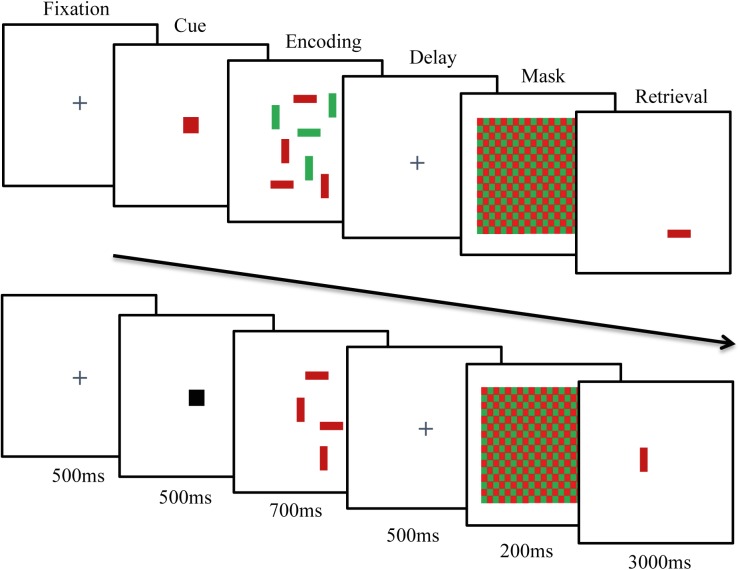
Schematic presentation of a *PC-DIIN* trial with a red cue for four red targets with four green distractors (upper row) and a trial with a black cue for four red targets without distractors (lower row).

The task was to memorize the horizontal or vertical orientation of red and green rectangles. Each trial started with a fixation cross followed by a cue (500 ms) indicating which color had to be encoded (Figure 1). In the no-distractor condition, a black cue indicated the presence of either only red or only green rectangles. A colored cue (red/green) indicated which rectangles were relevant to memorize (target) while they were embedded among distracting stimuli of the other color. The encoding display was presented for 700 ms. A 500 ms long delay was followed by a masking red-green checkerboard (200 ms) to prevent visual after images. Afterward, one of the targets was presented again on the screen. Participants had to decide whether this rectangle changed its orientation and responded by left or right button press within 3 s.

The task was executed with Matlab (R2013a, MathWorks, Inc.) and the Psychtoolbox Version 3.0.12 on a 15-inch screen with a resolution of 1024 × 1768. The display covers 4° × 9.3° angle of sight. The stimuli (0.28° × 0.72°) were placed on predetermined positions 1.79° to the left and to the right of the central fixation cross on a gray background.

The whole task contained 144 trials and lasted about 15 min. The set size varied from 4 to 6 targets. In distractor trials, the same amount of distractors was added (Schmicker et al., 2016). Each set size was presented 24 times without distractors and another 24 times with an equal amount of targets and distractors. Half of the trials were presented with red rectangles as targets whereas the other half consisted of green rectangles targets. The trial progression continued automatically.

#### Reality-Related Memory Room (*realDIIN*)

**FIGURE 2:**
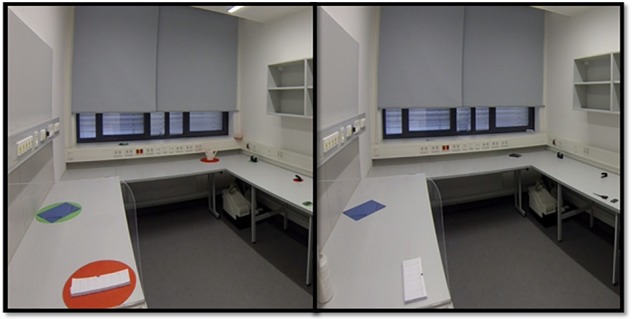
Example for the *realDIIN* room with six red targets and six green distractors; left picture: pan of the room during encoding time; right picture: same part of the room in the recognition phase when the colored circles were removed and the objects were changed.

To assess memory performance and *DIIN* effects in a realistic test setting we designed the *realDIIN* paradigm. Several objects had to be memorized in a prepared room. Because it has already been shown that difficulty increases with context compatible objects (Hollingworth et al., 2001) the room contained only items that can normally be found in an ordinary office (folder, calculator, scissors, marker, stapler, calendar, notebook, tape, watering can, alarm clock, lunch box, mug). Too large and heterogeneous objects were excluded to guarantee an appropriate level of difficulty. Finally, the twelve objects mentioned above were chosen as they fulfilled these criteria. All items were marked by color by standing on red or green cardboard circles. The circles had an adapted diameter of 133% of the longest side of an object. An exemplary trial is illustrated in Figure 2.

At the beginning of each trial, participants had to close their eyes and were told which objects had to be memorized (memory condition with distractors: only the red ones or only the green ones, memory condition without distractors: all marked objects; there is only one color). The examiner led them into the center of the room and asked them to open their eyes. The task was to encode the exact position and orientation of maximal six target objects in the testing room within 15 s. Afterward, the examiner asked them to close their eyes and led them out of the room. The examiner removed all the colored circles and changed the position or orientation of certain defined objects. After a retention interval of 1 min, the participant entered the room again and decided whether there was a change, which objects had changed and what kind of change had occurred, either in orientation or in position. The next instruction was to put the changed objects back to their former positions. Participants had unlimited time to do so. Between two trials the examiner prepared the next room arrangement, meanwhile the participant answered questionnaires.

As mentioned, there were two different kinds of changes: either orientation changes or position changes, but not both types of change at the same time on an individual object. A position change referred to an object that varied its position in the room for at least the same length of its colored circle’s diameter. An orientation change was characterized by turning around for 45°–90°. The number of changed objects per trial in the non-distracting and distracting condition varied from 0 to 6: one trial without any changes, one with one change, another one with five changes, one with six changes and two trials with three changes. Measured answers only accounted for the number of recognized changes, but not type of changes.

In total, each participant had to complete twelve trials: six trials without any distractors (6 objects per trial) and six trials with six targets and six additional distractors (12 objects per trial). The target color and the function of an object (target or distractor) varied systematically. To balance the effects of trial order, we offered three different orders and distributed them in a randomized way to the participants. The time intervals were chosen according to Hollingworth (2006).

#### Paper-Pencil Task (*CityMap*)

**FIGURE 3:**
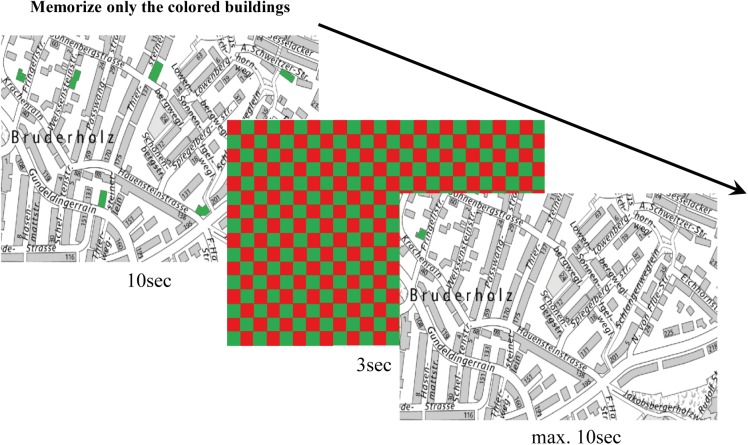
Example for a *CityMap* trial with six targets without distractors.

As *PC-DIIN* and *realDIIN* differ in many aspects, we decided to develop a paper-pencil task that is in between concerning closeness to everyday life, the degree of technology as well as motoric and perceptual demands. In this task, the participant had to memorize the orientation of marked buildings on a map (Figure 3).

The map excerpts of Basel were taken from the website Geoportal Kanton Basel-Stadt (o. D.^2^), were edited with Adobe Photoshop (Version CS 5. 1; Adobe Systems, 1990) and standardized in the width to 15 cm and in the mean height to 15 cm. We decided to use a real map instead of an invented one in order to make it look as realistic as possible. The whole material was presented in a file which was presented to each participant together with the answer sheet. In total, the task consisted of 24 trials with each presenting a different excerpt of the map of Basel.

In the *CityMap* task participants had to either memorize the orientation of all marked buildings (no distractors, black cue) or to memorize only one-colored buildings (e.g., red) while ignoring buildings of the other color (e.g., green, distractor condition). The cue (black, green or red) was presented for 1 s. Afterward, participants were asked to turn the page and to encode the colored buildings for 10 s. The presentation of the map was followed by a colored mask (1 s) similar to the one in the *PC-DIIN*. Afterward, the map was presented again, but only one of the previously marked buildings was colored. Participants had to decide whether the orientation of this marked building had changed. The examiner asked to turn the page over after 3 s (measured with a stopwatch). The tested object was turned for approximately 90° without disturbing the outline of the streets. Task difficulty varied with set size: In no-distractor conditions, 4–6 buildings had to be encoded while in the distractor condition 8, 10, or 12 buildings were marked. Half of the marked objects were targets, the other half were distractors (4+4, 5+5, 6+6). Each participant completed 24 trials with a 1-min break after half of the trials. To avoid effects of order we prepared four different randomized orders.

#### Questionnaires

A self-developed questionnaire was used to asses motivation of participants and their subjective evaluation of all three tasks. After completion of all three tasks, participants had to answer the following questions by choosing *PC-DIIN*, *CityMap*, or *realDIIN*: *In your opinion, which task did you perform best? Which of all three tasks would you rate as the most difficult one? Which task would you define as the most relevant in everyday life? Which task would you continue for further 30 min? Which task did you enjoy most?* They were also asked to give reasons and report strategies or thoughts about the tasks.

### Data Analysis

Data analysis was carried out with SPSS Version 21.0 (IBM SPSS Statistics, 2012). Performance was calculated as percentages of correct answers (correct rejections and hits) and reaction times (RT) in ms in the case for *PC-DIIN*. For analyzing *PC-DIIN* data we eliminated trials in which no responses occurred. First, this procedure should enhance the equivalence to *realDIIN* concerning the unlimited time interval for responses. Second, another study of ours had shown that older adults had been struggling with the computer-based task and reached scores near chance level including trials without any reactions. In addition, it seemed to be more sensible to exclude those trials disadvantaging older participants with sensory and motoric deceleration. In younger adults, 1% of all trials without distractors were excluded, in older adults 2.2%. Excluded distractor-trials in younger adults amounted to 0.8% and in older adults to 1.7%.

The assumption of sphericity was checked by using the Mauchly test and variance homogeneity was analyzed by using the Levene test. The normal distribution of all sub-conditions was tested with the Shapiro–Wilk test. The assumption of normal distribution could not be verified for the *CityMap* data. Therefore, we used non-parametric tests for analyzing these results.

We calculated 2 × 2 repeated-measures analysis of variances (ANOVAs) with the factors *age group* (younger adults, older adults) and *condition* (without distractors, with distractors) for each task separately. Dependent and independent *post hoc t*-tests, Mann–Whitney *U*-tests or Wilcoxon tests were used (Bonferroni corrected).

The *DIS* scores were calculated for each task: the percent correct in conditions with distractors (*Dis*) was subtracted from the one without distractors (*NoDis*) in each task. More negative *DIS* scores therefore reflected the ability to perform better in the presence of distractors.

To compare the task performances as well as the *DIS* scores over all tasks (within-subject factor *task type*: *PC-DIIN*, *realDIIN*, *CityMap*) and across both age groups (between-subject factor *age group*: younger, older), we conducted repeated-measures ANOVAs for performance and *DIS*-score.

Furthermore, Pearson and Spearman correlations were calculated to examine the relations between the three tasks, the *MoCA* and years of education. The level of significance was set to α = 0.05. We report standard deviations and effect sizes were interpreted according to Cohen (1992). In order to evaluate the effect sizes, we stated partial eta-squares ($\eta_{p}^{2}$) in ANOVA’s, Cohen’s *d* in *t*-tests and the correlation coefficients in non-parametric Wilcoxon tests, Mann–Whitney *U*-tests and correlations.

## Results

### Task-Specific Memory and Distractor Performance

**FIGURE 4:**
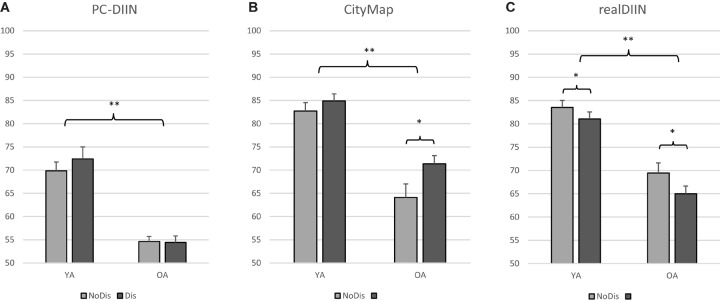
Performance for the *PC-DIIN* task **(A)**, the *CityMap* **(B)** and the *realDIIN* room **(C)**. Mean total percent correct and standard errors for trials without (bright, *NoDis*) and with distractors (dark, *Dis*) in both age groups (YA, younger adults: *n* = 27, 19–29 years; OA, older adults: *n* = 22, 61–77 years; ^∗^*p* < 0.05; ^∗∗^*p* < 0.001).

Test performances for younger and older adults in the conditions with and without distractors in *PC-DIIN*, *CityMap*, and *realDIIN* are visualized in Figure 4.

#### PC-DIIN

A repeated-measures ANOVA revealed a significant main effect in performance (% correct) for *age group* [*F*(1,47) = 43.32, *p* < 0.001, $\eta_{p}^{2}$ = 0.480]. Younger adults outperformed older adults as they reached 16.6% more percent correct (Figure 4A). For *PC-DIIN* performance, there was no main effect for *condition* (*p* = 0.305) and no interaction effect (*p* = 0.226).

In addition, older adults responded significantly slower than the younger ones [YA: *M* = 1119 ms, *SD* = 0.187; OA: *M* = 1420 ms, *SD* = 0.494, *t*(47) = −5.04, *p* < 0.001, *d* = −1.448]. A speed accuracy tradeoff was only found for older adults. They improved their test performance in *PC-DIIN* when they performed more slowly [YA: *r*(25) = 0.216, *p* = 0.280; OA: *r*(20) = 0.516, *p* = 0.014].

Young adults spent 10 h more on the computer compared to older adults (*p* < 0.005). But there was no significant correlation between the number of hours spent on the computer per week and the results in the *PC-DIIN* for younger and older adults [*r*_y_(25) = −0.126, *p* = 0.532; *r*_o_(18) = 0.287, *p* = 0.220].

#### CityMap

Significant main effects for *age group* [*F*(1,48) = 55.55, *p* < 0.001, $\eta_{p}^{2}$ = 0.538] and for *condition* [*F*(1,48) = 6.24, *p* = 0.016, $\eta_{p}^{2}$ = 0.115] were found in a repeated-measures ANOVA for *CityMap* performances (Figure 4B). No interaction effect (*p* = 0.183) was observed. Younger participants reached 16.05% more percent correct in all *CityMap* trials. All subjects showed 4.5% more correct answers in the condition with distractors. This difference was only significant for older adults (Wilcoxon: *Z* = −2.38, *p* = 0.018).

#### realDIIN

A repeated-measures ANOVA revealed significant main effects on *realDIIN* performance for *age group* [*F*(1,48) = 53.60, *p* < 0.001, $\eta_{p}^{2}$ = 0.528] and *condition* [*F*(1,48) = 21.41, *p* < 0.001, $\eta_{p}^{2}$ = 0.308]. Younger adults performed 15.09% better than older adults. For the no-distraction condition (NoDis) the overall performance of all subjects was 6.3% higher compared to the condition including distractors (Dis) (Figure 4C). *T*-tests revealed significant differences between NoDis and Dis for both younger adults [*t*(26) = −3.405, *p* = 0.002] and older adults [*t*(22) = −3.141, *p* = 0.005].

### Performance Over Task Type and Age

The repeated-measures ANOVA revealed a significant effect of *age-group* [*F*(1,47) = 149.27, *p* < 0.001, $\eta_{p}^{2}$ = 0.761] and of *task type* on the overall performance [*F*(2,94) = 48.33, *p* < 0.001, $\eta_{p}^{2}$ = 0.507]. All participants performed significantly worse in *PC-DIIN* than in *CityMap* [*F*(1,47) = 81.44, *p* < 0.001, $\eta_{p}^{2}$ = 0.634] and *realDIIN* [*F*(1,47) = 59.76, *p* < 0.001, $\eta_{p}^{2}$ = 0.560]. On the other hand, performances in *realDIIN* and *CityMap* did not differ significantly [*t*(49) = −0.821, *p* = 0.416, *d* = −0.164]. An interaction effect *age ×task type* was not observed in the total percent correct [*F*(2,94) = 0.05, *p* = 0.956, $\eta_{p}^{2}$ = 0.001].

### DIS Scores

**FIGURE 5:**
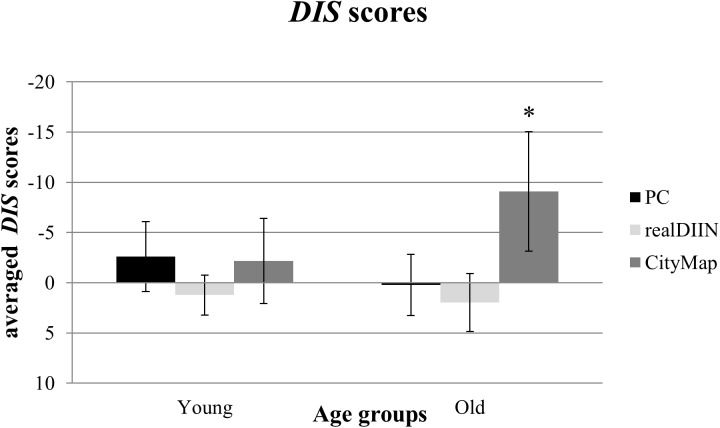
*DIS* scores of both age groups (young = 19–29 years; old = 61–77 years) in all three test paradigms with a 95%-confidence interval (^∗^*p* < 0.05).

To examine the influence of *task type* and *age group* on distractor effects we ran a repeated-measures ANOVA on the *DIS* scores (NoDis – Dis). There was no significant main effect for *age group* [*F*(1,47) = 0.76, *p* = 0.387, $\eta_{p}^{2}$ = 0.016] but a significant main effect of *task type* [*F*(2,94) = 7.23, *p* = 0.002, $\eta_{p}^{2}$ = 0.133]. Furthermore, the *task type* influenced the performance differently depending on the age-group [interaction effect: *F*(2,94) = 3.58, *p* = 0.036, $\eta_{p}^{2}$ = 0.071] (Figure 5). An one-way ANOVA revealed a significant *task type* effect for older adults [*F*(2,42) = 7.84, *p* < 0.001, $\eta_{p}^{2}$ = 0.272] indicating higher negative *DIS* scores in *CityMap* (*p* < 0.05). No significant *task type* effect was found in younger adults.

Overall, participants reached higher negative *DIS* scores in *CityMap* than in *realDIIN* (*Z* = −2.66, *p* = 0.008, *r* = −0.376), whereas *PC-DIIN* and *CityMap* (*Z* = −1.59, *p* = 0.113, *r* = −0.227) as well as *PC-DIIN* and *realDIIN* did not differ significantly [*t*(48) = −1.96, *p* = 0.056, *d* = −0.396]. Higher negative *DIS* scores in *PC-DIIN* and *CityMap* reflect that participants performed better in the presence of distractors.

The significant interaction effect means that older adults performed better than younger ones in conditions with distractors in the *CityMap*. The contrast between age groups did not reach significance (Kolmogorov–Smirnov *Z* = 1.27, *p* = 0.079), but showed a tendency for higher *DIS* scores in older adults.

### Motivation and Evaluation

Questionnaire data for motivation and evaluation ratings can be found in Table 1.

**Table 1:** Frequency of marked tasks in the motivation and evaluation questionnaire in %.

	Performance		Difficulty		Everyday relevance		Continuation		Enjoy	
	YA	OA	YA	OA	YA	OA	YA	OA	YA	OA
*PC-DIIN*	0	0	81.5	73.9	0	0	0	0	0	0
*CityMap*	44.4	82.6	7.4	0.0	18.5	26.1	33.3	47.8	22.2	39.1
*realDIIN*	55.6	13.0	11.1	21.7	81.5	65.2	66.7	47.8	77.8	56.5

*Twenty seven younger adults (YA) and 23 older adults (OA) answered the questions “In your opinion, which task did you perform best?” (Performance) “Which of all three tasks would you rate as the most difficult one?” (Difficulty) “Which task would you define as the most relevant in everyday life?” (Everyday relevance) “Which task would you continue for further 30 min?” (Continuation) and “Which task did you enjoy most?” (Enjoy).*

Older adults reported *CityMap* as the subjectively best performed task, whereas younger participants reported *realDIIN* 11.2% more often. All participants evaluated *PC-DIIN* as the most difficult one and *realDIIN* as the task with the subjectively highest relevance for everyday life and the task that was most enjoyable. Whereas older adults would continue *CityMap* and *realDIIN* with the same frequency, younger adults would prefer *realDIIN* for a continuation. All in all, *PC-DIIN* was the least favored and the most difficult rated task.

Subjects stated that *PC-DIIN* was difficult due to fast speed, monotony, no breaks, producing fatigue, being far from reality and attentional decrease. *realDIIN* was evaluated as directly experienced/real, not monotonous, diverse in perspectives, easier due to landmarks and contextual support, optimal in given processing time, close to reality. For the *CityMap* subjects reported that it was easy to orient, the test duration was optimal, they were less distracted and compared to *realDIIN* the short delay time was rated as more comfortable.

Participants were asked for utilizing strategies. Both younger and older adults reported a number of strategies in all three tasks. They formed global structures, grouped the stimuli to meaningful figures or used prominent attributes of stimuli (*CityMap* and *realDIIN*). Especially older adults often used the strategy to memorize buildings in the *CityMap* relative to the streets and other contextual landmarks.

In the questionnaire, we also assessed whether participants were biased by knowing Basel to control for respective familiarity. However, none of the participants stated to have good knowledge about the city.

### Correlations

**FIGURE 6:**
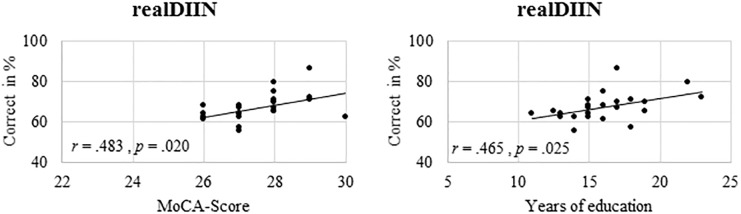
Older participants test performances in *realDIIN* in relation to their *MoCA* scores and to their years of education.

The three tasks did not correlate significantly with one another. Furthermore, the relation between all three tasks, years of education and the *MoCA* value was analyzed. *MoCA* and education served as indicators for cognitive health and neuroprotective factors. There were no significant correlations for younger adults. In the older adults, however, *realDIIN* correlated with the scores of the *MoCA* and the years of education. Yet, *MoCA* and years of education did not correlate with *PC-DIIN/CityMap*. The significant correlations are visualized in Figure 6. All correlations can be found in the Supplementary Material (Supplementary Table S1).

## Discussion

The present study investigated change-detection memory performance and attentional control, namely distractor processing, in younger and older adults over different test settings that vary regarding the closeness to reality: a computer task (*PC-DIIN*), a paper-pencil (*CityMap*) and a real room (*realDIIN*).

### Performance in Each Task

Younger participants outperformed the older ones in each test. This finding matches general statements of age-related research that explains age-differences by a cognitive decline in attention, memory, associations, perceptual, and processing speed (Rhodes, 2004; Mienaltowski, 2011). In the *PC-DIIN* where trials were time limited, older adults performed with near-to-chance performance, which could be a sign for being overwhelmed (Salthouse, 2000). Indeed, older adults responded more correctly when they reduce their reaction times in *PC-DIIN*. On the one hand, this speed-accuracy-tradeoff can support the assumption that processing speed is generally reduced in older adults (Salthouse, 2000, 2001). On the other hand, this finding could reflect the endeavor to compensate for low performance and thus could give evidence for strategy development using cognitive reserves of executive control (Stern, 2002).

The expected distractor effect only appeared in *realDIIN* as younger and older adults became worse under distraction. In contrast, the differences between age groups in the *CityMap* were due to a better performance in the presence of distractors in older but not in younger adults. In the *PC-DIIN*, distractors did not influence performances of our subjects. Significant interaction effects for the *DIS* score will be discussed later.

As expected, older adults do not have as much computer experience as younger adults. These differences do not relate to the performance in *PC-DIIN* going in line with Jeong (2012), where participants with more exposure to and experiences with computers are not advantaged compared to non-experienced adults.

### Influence of Closeness to Reality on Age-Related Memory Performance

All participants showed significantly lower test performances in the *PC-DIIN* compared to the *realDIIN* and the *CityMap*. However, the expected improvement of performance with increasing degree of closeness to reality failed to appear: the *realDIIN* test did not produce the most correct answers. Furthermore, older adults did not benefit from more realistic test scenarios.

Why did the third dimension and additional vestibular, proprioceptive, and kinesthetic information not influence performance like former studies (Montello et al., 2004; Piccardi et al., 2008) have claimed? Cockburn and McKenzie (2002) reported that a three-dimensional test setting is not automatically accompanied by reduced task difficulty. Real situations can be complex and confusing, which leads to a growing cognitive demand. In *realDIIN* the test setting was also characterized by a higher complexity and the difficulty could be manipulated through a higher number of encoding information. Movements, searching, exploring the targets’ positions and orientations and developing strategies require many cognitive capacities. In addition, targets and distractors had semantic properties and came from the same object pool enlarging the interference between the trials. The difficulty was hence increased as the task involved context compatible items (Hollingworth et al., 2001). These additional demands could prevent benefits in comparison to the 2D *CityMap*. The explanation for worse performance in *realDIIN* could thus simply refer to the extent of the complex visual scene.

Besides, the results in general memory performance did not show any reduction due to age-related differences. There is no compensation for decreased performance in older adults as it was claimed by Cherry and Park (1993) and Mazurek et al. (2015). The absence of this *age group × task type* interaction in overall percent correct contradicts the assumption that older adults may be disadvantaged by the use of computers when estimating their cognitive abilities.

### Using Distractors for Compensating Age-Related Decline

Surprisingly, distractors led to significantly better memory performance in older adults in the *CityMap* test as the significant interaction *age group × task type* for the *DIS* score revealed. This result reflects the age-related compensation effect, as it was claimed for the general performance before. Although older adults’ general memory performance has not been facilitated by more realistic test scenarios, they benefited more from distractors in this paper-pencil task compared to younger adults.

Here, it is important to consider three advantageous factors of this paper-pencil task. First, the complexity of the stimulus material does not require such a cognitive effort as *realDIIN* does. Second, *CityMap* provides environmental context information (buildings are surrounded by streets, other houses and parks) for possible strategies. Third, the encoding interval was long enough to process all target buildings. Whereas in *PC-DIIN* the processing time was very short, participants had more time for memorizing the *CityMap*.

We assume that older adults need additional time (like *CityMap* provides) to encode distractors in addition to targets and use them as reference points. Therefore, distractors may act as a kind of help that, together with contextual information, encourage memory strategies in older adults. Older adults are known to increasingly use distractors as an environmental support in dependence of the task structure (Lindenberger and Mayr, 2014). In this manner, compensation may be explained by the development of mechanic and pragmatic intelligence in the course of life (Baltes, 1997). This finding goes in line with the stated late filtering in older adults (Jost et al., 2010). While younger adults can handle the tasks quite well using cognitive skills like executive attention, memory and processing speed, older adults have to search for alternative processing to counterbalance their decreasing cognitive performance (Stern, 2002). Older adults reported to memorize the marked buildings in the *CityMap* with the support of environmental landmarks like global patterns, building structures, location relative to the streets or other surrounding information of the map.

Obviously, *CityMap* offers the possibility to invest more effort and time and to develop a new skill (using environmental support) in order to compensate for less performance (Dixon et al., 2001). As it was shown that older adults compensate for memory decline more successfully when they have higher cognitive reserve available (Frankenmolen et al., 2018), we suggest that cognitive reserve could play an important role in using environmental support for increasing their performance.

### Advantages of More Ecologically Valid Tasks

*PC-DIIN* was subjectively rated as most difficult and unpopular and *CityMap* was rated as the easiest task. Both failed to correlate to other measures. As the *PC-DIIN* performance in older adults was at chance level it is not surprising that we found no correlation with years of education or *MoCA*. If the computer version measured above floor performance, it would potentially correlate with the PC task performance. However, *realDIIN* test was the most favored test of all and displayed a relationship to a cognitive screening measurement, the *MoCA*, and years of education. The *MoCA* measures multiple cognitive domains and may also be useful as an indicator for general cognitive abilities. The observed correlation may indicate that participants with better performance in a real environment have more general cognitive abilities available (Garrett et al., 2010) and use memory strategies acquired during learning and educational periods. This result might also be evident in the relationship between reality-based performance in *realDIIN* and everyday behavior. As education is known as a protective factor against cognitive decline and dementia (Evans et al., 1993; Ball and Birg, 2002) and can be seen as a predictor for strategy use (Garrett et al., 2010) we consider *realDIIN* as a first step to concentrate on more realistic test scenarios.

In order to support this assumption between *realDIIN* and everyday behavior, we suggest collecting more cognitive data (e.g., using the Memory Compensation Questionnaire (MCQ), Dixon et al., 2001). Anyway, performance in the computerized task is hardly able to be generalized to everyday-related behavior as it measures internally valid factors, e.g., the ability to deal with abstract stimulus material under high time pressure.

### General Limitations

There are several limitations that have to be mentioned in the end. It has to be taken into account that these results are based on a small sample and that there was a quite limited number of trials, especially for *realDIIN* and *CityMap*. No correlations were found between the conducted tests in either group. As stated before, it is actually not appropriate to directly compare internally valid tests with ecologically valid tests. The claim to develop more realistic test scenarios holds the risk of actually measuring different underlying constructs. Moreover, it is very costly and not possible to realize equal test settings when creating corresponding real, paper-pencil and computer versions of one task. Using the same number of trials in *realDIIN*, for example, would result in a multiple hour testing session. To make artificial stimulus material more realistic it is also important to use real objects, which of course are different from vertical or horizontal bars or colored buildings. We deliberately decided to enhance the processing time in the more ecologically valid tasks because we were aware of the greater amount of information included in real sceneries like rooms or maps. Moreover, as the manipulation of the objects in the real room took more time than a computerized direction change of a bar, we had no choice but to extend the retention interval for practical reasons.

Furthermore, the three tests obviously do not deal with the same kind of memory. Whereas the *PC-DIIN* task measures working memory both other tasks have longer encoding and maintenance intervals. Besides the closeness to reality, the methods differed systematically in their time course, trial number, response type, stimulus material, in their demand on motoric and perceptual speed and their availability of contextual encoding information and strategies. Therefore, more adaptions of the *DIIN* task are required, i.e., a computerized task with real-world objects or a real task with abstract shapes. There are efforts to design laboratory visual search tasks that allow testing attention and memory performance in real-world behavior, e.g., airport security and medical screening in a controlled way (Evans et al., 2013; Wolfe et al., 2013) and recently also to test age differences therein (Wiegand and Wolfe, 2018). These approaches should be taken into account when developing comparable tasks in order to answer questions about ecological validity. Therefore, we would suggest handling our study design as a preliminary stage of a prospective investigation that will be adapted to stricter criteria. Albeit, we showed that more realistic test settings influence age-related cognitive control processes which therefore lead to an advantageous distractor processing in older adults under particular circumstances.

## Conclusion

Our findings do not support the general notion that older adults would profit from more realistic test scenarios. In spite of missing general interactions regarding age and type of testing, we found a systematic effect of age depending on the closeness to reality for attentional mechanisms in memory performance (*DIS* score). In contrast to previous findings, the presence of task-irrelevant distractors actually helped to improve older adults’ performance in the paper pencil task arguing against the assumption of a general age-related impairment of inhibition.

Moreover, performance in a clinical screening task correlated only with the performance in the real world task suggesting that this task reflected the general cognitive status of participants better than the more abstract tasks. This task also causes higher test motivation, promising stronger commitment of participants. This is an important requirement for cognitive training designs (Schmicker et al., 2016), higher training outcomes and probably better transfer effects (Colquitt et al., 2000; Prins et al., 2011).

In sum, the present results show that age-related changes in memory are neither simply explained by reduced abilities to deal with abstract computer tasks nor by disturbed inhibition processes. Apparently, a responsible psychological approach that claims to investigate cognitive mechanisms in higher age should focus on the population’s needs for real, complex tasks and the possibility to use compensational strategies according to the cognitive reserve. Future studies have to find a way to retain advantages and reduce disadvantages in realistic testing. One possibility could lay in virtual reality settings (Parsons, 2015) as this method reaches high degrees of ecological validity but is more convenient than realistic tests (Grewe et al., 2014). It would also be very important to show that older adults use their cognitive reserve to handle cognitive tasks more successfully compared to younger adults.

## Ethics Statement

All subjects gave written informed consent in accordance with the Declaration of Helsinki. The protocol was approved by the “ethics committee of Magdeburg.”

## Author Contributions

MS and UR developed the idea for this study, designed the tasks, and drafted the manuscript. UR collected and analyzed the data. IM, MS, and NM contributed to the discussion of content-related issues and to the critical revision of the article.

## Conflict of Interest Statement

The authors declare that the research was conducted in the absence of any commercial or financial relationships that could be construed as a potential conflict of interest.

## Footnotes

1 Concerning the term distractor inhibition, we have to note that additional stimuli are not instructed to attend and, therefore, were expected to “distract.” Indeed, the distractors in our study did not influence the performance of our participants in the way we had expected. As it would be confusing to rename we will keep this wording while emphasizing that the original meaning of distractors should not be over interpreted in our tasks.

2 https://map.geo.bs.ch

## References

[B1] Aberle I. Rendell P. G. Rose N. S. McDaniel M. A. Kliegel M. (2010). The age prospective memory paradox: young adults may not give their best outside of the lab. *Dev. Psychol.* 46:1444. 10.1037/a0020718 21058832 PMC3071572

[B2] Bäckman L. (1985). Compensation and recoding: a framework for aging and memory research. *Scand. J. Psychol.* 26 193–207. 4070994 10.1111/j.1467-9450.1985.tb01157.x

[B3] Ball L. J. Birg S. J. (2002). Preventions of brain aging and dementia. *Clin. Geriatr. Med.* 18 485–503. 12424869 10.1016/s0749-0690(02)00027-7

[B4] Baltes P. B. (1997). On the incomplete architecture of human ontogeny: selection, optimization, and compensation as foundation of developmental theory. *Am. Psychol.* 52 366–380. 9109347 10.1037//0003-066x.52.4.366

[B5] Chen J. Hale S. Myerson J. (2003). Effects of domain, retention interval, and information load on young and older adults’ visuospatial working memory. *Aging Neuropsychol. Cogn.* 10 122–133.

[B6] Cherry K. E. Park D. C. (1993). Individual difference and contextual variables influence spatial memory in younger and older adults. *Psychol. Aging* 8 517–526. 8292280 10.1037//0882-7974.8.4.517

[B7] Cockburn A. McKenzie B. (2002). “Evaluating the effectiveness of spatial memory in 2D and 3D physical and virtual environments,” in *Proceedings of the SIGCHI Conference on Human Factors in Computing Systems*, eds Grinter R. Rodden T. Aoki P. Cutrell E. Jeffries R. Olson G. (New York, NY: Association for Computing Machinery), 203–210.

[B8] Cohen G. (1989). *Memory in the Real World.* Hillsdale: Lawrence Erlbaum Associates.

[B9] Cohen J. (1992). A power primer. *Psychol. Bull.* 112 155–159. 19565683 10.1037//0033-2909.112.1.155

[B10] Colquitt J. A. LePine J. A. Noe R. A. (2000). Toward an integrative theory of training motivation: a meta-analytic path analysis of 20 years of research. *J. Appl. Psychol.* 85 678–707. 11055143 10.1037/0021-9010.85.5.678

[B11] Craik F. I. Bialystok E. (2006a). Cognition through the lifespan: mechanisms of change. *Trends Cogn. Sci.* 10 131–138. 16460992 10.1016/j.tics.2006.01.007

[B12] Craik F. I. Bialystok E. (2006b). Planning and task management in older adults: cooking breakfast. *Mem. Cogn.* 34 1236–1249. 17225505 10.3758/bf03193268

[B13] Craik F. I. M. (1994). Memory changes in normal aging. *Curr. Dir. Psychol. Sci.* 3 155–158.

[B14] Craik F. I. M. Byrd M. (1982). “Aging and Cognitive Deficits,” in *Aging and Cognitive Processes. Advances in the Study of Communication and Affect* Vol. 8 eds Craik F. I. M. Trehub S. (Boston, MA: Springer).

[B15] Crawford S. Channon S. (2002). Dissociation between performance on abstract tests of executive function and problem solving in real-life-type situations in normal aging. *Aging Ment. Health* 6 12–21. 11827618 10.1080/13607860120101130

[B16] Czaja S. J. Charness N. Fisk A. D. Hertzog C. Nair S. N. Rogers W. A. (2006). Factors predicting the use of technology: findings from the center for research and education on aging and technology enhancement (CREATE). *Psychol. Aging* 21 333–352. 16768579 10.1037/0882-7974.21.2.333 PMC1524856

[B17] Dixon R. A. de Frias C. M. Bäckman L. (2001). Characteristics of self-reported memory compensation in older adults. *J. Clin. Exp. Neuropsychol.* 23 650–661. 11778642 10.1076/jcen.23.5.650.1242

[B18] Eid M. Gollwitzer M. Schmitt M. (2010). *Statistik und Forschungsmethoden.* Weinheim: Beltz-Verlag.

[B19] Evans D. A. Beckett L. A. Albert M. S. Hebert L. E. Scherr P. A. Funkenstein H. H. (1993). Level of education and change in cognitive function in a community population of older persons. *Ann. Epidemiol.* 3 71–77. 8287159 10.1016/1047-2797(93)90012-s

[B20] Evans K. K. Birdwell R. L. Wolfe J. M. (2013). If you don’t find it often, you often don’t find it: why some cancers are missed in breast cancer screening. *PLoS One* 8:e64366. 10.1371/journal.pone.0064366 23737980 PMC3667799

[B21] Frankenmolen N. L. Overdorp E. J. Fasotti L. Claassen J. A. Kessels R. P. Oosterman J. M. (2018). Memory strategy training in older adults with subjective memory complaints: a randomized controlled trial. *J. Int. Neuropsychol. Soc.* 24 1110–1120. 30168408 10.1017/S1355617718000619 PMC6317111

[B22] Garrett D. D. Grady C. L. Hasher L. (2010). Everyday memory compensation: the impact of cognitive reserve, subjective memory, and stress. *Psychol. Aging* 25:74. 10.1037/a0017726 20230129

[B23] Grewe P. Lahr D. Kohsik A. Dyck E. Markowitsch H. J. Bien C. G. (2014). Real-life memory and spatial navigation in patients with focal epilepsy: ecological validity of a virtual reality supermarket task. *Epilepsy Behav.* 31 57–66. 10.1016/j.yebeh.2013.11.014 24361763

[B24] Hedden T. Gabrieli J. D. (2004). Insights into the ageing mind: a view from cognitive neuroscience. *Nat. Rev. Neurosci.* 5:87. 14735112 10.1038/nrn1323

[B25] Hollingworth A. (2006). Scene and position specificity in visual memory for objects. *J. Exp. Psychol.* 32 58–69. 10.1037/0278-7393.32.1.58 16478340

[B26] Hollingworth A. Williams C. C. Henderson J. M. (2001). To see and remember: visually specific information is retained in memory from previously attended objects in natural scenes. *Psychon. Bull. Rev.* 8 761–768. 11848597 10.3758/bf03196215

[B27] Jeong H. (2012). A comparison of the influence of electronic books and paper books on reading comprehension, eye fatigue, and perception. *Electron. Library* 30 390–408.

[B28] Johnson M. K. McMahon R. P. Robinson B. M. Harvey A. N. Hahn B. Leonard C. J. (2013). The relationship between working memory capacity and broad measures of cognitive ability in healthy adults and people with schizophrenia. *Neuropsychology* 27 220–229. 10.1037/a0032060 23527650 PMC3746349

[B29] Jost K. Bryck R. L. Vogel E. K. Mayr U. (2010). Are old adults just like low working memory young adults? Filtering efficiency and age differences in visual working memory. *Cereb. Cortex* 21 1147–1154. 10.1093/cercor/bhq185 20884722

[B30] Lindenberger U. Mayr U. (2014). Cognitive aging: is there a dark side to environmental support? *Trends Cogn. Sci.* 18 7–15. 10.1016/j.tics.2013.10.006 24210962 PMC3969029

[B31] Lustig C. Hasher L. Zacks R. T. (2007). Inhibitory deficit theory: recent developments in a “new view”. *Inhib. Cogn.* 17 145–162.

[B32] Mazurek A. Bhoopathy R. M. Read J. C. Gallagher P. Smulders T. V. (2015). Effects of age on a real-world what-where-when memory task. *Front. Aging Neurosci.* 7:74. 10.3389/fnagi.2015.00074 26042030 PMC4435419

[B33] Mienaltowski A. (2011). Everyday problem solving across the adult life span: solution diversity and efficacy. *Ann. N. Y. Acad. Sci.* 1235 75–85. 10.1111/j.1749-6632.2011.06207.x 22023569 PMC3746011

[B34] Montello D. R. Waller D. Hegarty M. Richardson A. E. (2004). “Spatial memory of real environments, virtual environments, and maps,” in *Human Spatial Memory: Remembering Where*, ed. Allen G. L. (Brighton: Psychology Press), 251–285.

[B35] Nasreddine Z. S. Phillips N. A. Bédirian V. Charbonneau S. Whitehead V. Collin I. (2005). The montreal cognitive assessment, MoCA: a brief screening tool for mild cognitive impairment. *J. Am. Geriatr. Soc.* 53 695–699. 15817019 10.1111/j.1532-5415.2005.53221.x

[B36] Naveh-Benjamin M. (2000). Adult age differences in memory performance: tests of an associative deficit hypothesis. *J. Exp. Psychol.* 26 1170–1187. 10.1037//0278-7393.26.5.1170 11009251

[B37] Noyes J. M. Garland K. J. (2008). Computer-vs. paper-based tasks: are they equivalent?. *Ergonomics* 51 1352–1375. 10.1080/00140130802170387 18802819

[B38] Parsons T. D. (2015). Virtual reality for enhanced ecological validity and experimental control in the clinical, affective and social neurosciences. *Front. Hum. Neurosci.* 9:660. 10.3389/fnhum.2015.00660 26696869 PMC4675850

[B39] Phillips L. H. Kliegel M. Martin M. (2006). Age and planning tasks: the influence of ecological validity. *Int. J. Aging Hum. Dev.* 62 175–184. 16541929 10.2190/EM1W-HAYC-TMLM-WW8X

[B40] Piccardi L. Iaria G. Ricci M. Bianchini F. Zompanti L. Guariglia C. (2008). Walking in the Corsi test: which type of memory do you need? *Neurosci. Lett.* 432 127–131. 10.1016/j.neulet.2007.12.044 18226450

[B41] Prins P. J. Dovis S. Ponsioen A. Ten Brink E. Van der Oord S. (2011). Does computerized working memory training with game elements enhance motivation and training efficacy in children with ADHD? *Cyberpsychol. Behav. Soc. Netw.* 14 115–122. 10.1089/cyber.2009.0206 20649448

[B42] Rendell P. G. Henry J. D. (2009). A review of virtual week for prospective memory assessment: clinical implications. *Brain Impair.* 10 14–22.

[B43] Rhodes M. G. (2004). Age-related differences in performance on the Wisconsin card sorting test: a meta-analytic review. *Psychol. Aging* 19 482–494. 15382998 10.1037/0882-7974.19.3.482

[B44] Salthouse T. A. (1994). The aging of working memory. *Neuropsychology* 8 535–543.

[B45] Salthouse T. A. (2000). Aging and measures of processing speed. *Biol. Psychol.* 54 35–54. 11035219 10.1016/s0301-0511(00)00052-1

[B46] Salthouse T. A. (2001). Attempted decomposition of age-related influences on two tests of reasoning. *Psychol. Aging* 16 251–263. 11405313 10.1037//0882-7974.16.2.251

[B47] Schmicker M. Müller P. Schwefel M. Müller N. G. (2017). Attentional filter training but not memory training improves decision-making. *Front. Hum. Neurosci.* 11:138. 10.3389/fnhum.2017.00138 PMC5362583 28386225

[B48] Schmicker M. Schwefel M. Vellage A. K. Müller N. G. (2016). Training of attentional filtering, but not of memory storage, enhances working memory efficiency by strengthening the neuronal gatekeeper network. *J. Cogn. Neurosci.* 28 636–642. 26765946 10.1162/jocn_a_00922

[B49] Stern Y. (2002). What is cognitive reserve? Theory and research application of the reserve concept. *J. Int. Neuropsychol. Soc.* 8 448–460. 11939702

[B50] Vogel E. K. Machizawa M. G. (2004). Neural activity predicts individual differences in visual working memory capacity. *Nature* 428 748–751. 15085132 10.1038/nature02447

[B51] Vogel E. K. McCollough A. W. Machizawa M. G. (2005). Neural measures reveal individual differences in controlling access to working memory. *Nature* 438 500–503. 16306992 10.1038/nature04171

[B52] Wästlund E. Reinikka H. Norlander T. Archer T. (2005). Effects of VDT and paper presentation on consumption and production of information: psychological and physiological factors. *Comput. Hum. Behav.* 21 377–394.

[B53] Wiegand I. Wolfe J. (2018). Hybrid visual and memory search is preserved in older age. *J. Vis.* 18 531–531. 31050319 10.1080/13825585.2019.1604941 PMC6825883

[B54] Wolfe J. M. Brunelli D. N. Rubinstein J. Horowitz T. S. (2013). Prevalence effects in newly trained aiport checkpoint screeners: trained observers miss rare targets, too. *J. Vis.* 13:33. 10.1167/13.3.33 24297778 PMC3848386

