# Supplementary material for: Investigating the Potential Role of Ecological Validity on Change-Detection Memory Tasks and Distractor Processing in Younger and Older Adults
Source: Front Psychol. 2019 May 24;10:1046. doi: 10.3389/fpsyg.2019.01046 (PMC6542998; doi:10.3389/fpsyg.2019.01046)
Supplement: Supplementary file 1 [file Table_1.pdf]

Supplementary Material

Table S1 Correlations

		PC	realDIIN	CityMap
YA	PC	-	-	-
	realDIIN	.349	-	-
	CityMap	.094	.238	-
	MAC-S	-.029	.155	-.147
	MoCA	-.027	.068	.181
	Years of Education	.032	.036	.137
OA	PC	-	-	-
	realDIIN	.261	-	-
	CityMap	-.183	.055	-
	MAC-S	-.127	-.252	-.297
	MoCA	.040	.483*	.353
	Years of Education	.220	.465*	-.001

YA…younger adults, OA…older adults; n = 50, nYA = 27, nOA = 23, for correlations with PC: n = 49, nYA = 27,nOA = 22, *p Wert < .05, **p < .001.
